# miR‐127‐5p negatively regulates enterovirus 71 replication by directly targeting SCARB2

**DOI:** 10.1002/2211-5463.12197

**Published:** 2017-04-13

**Authors:** Chunhong Feng, Yuxuan Fu, Deyan Chen, Huanru Wang, Airong Su, Li Zhang, Liang Chang, Nan Zheng, Zhiwei Wu

**Affiliations:** ^1^ Center for Public Health ResearchMedical SchoolNanjing UniversityChina; ^2^ School of life sciencesNanjing UniversityChina; ^3^ State Key Lab of Analytical Chemistry for Life ScienceNanjing UniversityChina; ^4^ Medical School and Jiangsu Key Laboratory of Molecular MedicineNanjing UniversityChina

**Keywords:** Enterovirus 71, miR‐127‐5p, SCARB2, viral replication

## Abstract

Enterovirus 71 (EV71) is the major causative agent of hand‐foot‐and‐mouth disease in young children and can cause severe cerebral and pulmonary complications and even fatality. This study aimed at elucidating whether and how EV71 infection is regulated by a cellular microRNA, miR‐127‐5p. We found that miR‐127‐5p can downregulate the expression of SCARB2, a main receptor of EV71, by targeting two potential sites in its 3′ UTR region and inhibit EV71 infection. Meanwhile, miR‐127‐5p expression was upregulated during EV71 infection. Notably, transfecting cells with miR‐127‐5p mimics led to a significant decrease in viral replication, while inhibition of endogenous miR‐127‐5p facilitated viral replication. Furthermore, our evidence showed that miR‐127‐5p did not affect postentry viral replication. Taken together, these results indicated that miR‐127‐5p inhibited EV71 replication by targeting the SCARB2 mRNA.

**Abbreviations**
CPE: cytopathic effect
EV71: Enterovirus 71
miRNAs: microRNAs
PSGL‐1: P‐selectin glycoprotein ligand‐1
SCARB2: scavenger receptor class B, member 2
UTR: untranslated region

MicroRNAs (miRNAs) are a class of highly conserved noncoding RNA oligonucleotides of 21–22 nucleotides that are found in animals, plants, and viruses 1, 2, 3. These small RNAs primarily regulate the expression of specific genes by binding to complementary sequences at target mRNAs to modulate their translation or stability. There are hundreds of miRNA‐encoding genes in humans, which regulate protein‐encoding genes 4, 5. Growing evidence has demonstrated that miRNAs regulate a wide range of biological processes, including development, differentiation, cell proliferation, apoptosis, and immune responses 1, 4.

Enterovirus 71 (EV71), a positive, single‐stranded RNA virus belonging to the family of Picornaviridae 6, 7, was first isolated from patients with neurological diseases, including fatal encephalitis and aseptic meningitis, in California in 1969 8. Later studies suggested that EV71 is associated with hand‐foot‐and‐mouth disease (HFMD) in young children and infants 9. In recent years, epidemic or sporadic outbreaks of neurovirulent EV71 infections have been reported in Southeast or East Asia, including Taiwan, Malaysia, Singapore, Japan, and China 10, 11, 12. EV71 infection has become a public health challenge. Although a vaccine has recently been approved in China, its clinical benefits remain to be seen.

Recently, miRNAs have been noted to be key effector molecules in the complex interaction network between virus and host 3, 13, either by targeting cellular factors used for virus replication 14, 15 or by directly targeting viral mRNAs 16, 17, 18. Plus‐strand RNA virus replication requires the recruitment of specific host factors at various steps in the process. These host factors help viral genomic replication, viral protein synthesis, and defense against host immune response 19.

Viral entry into host cells during infection is initiated by receptor‐mediated endocytosis involving specific cellular surface components 20. In the previous studies, a number of molecules have been identified to be functional receptors for EV71, including scavenger receptor class B, member 2 (SCARB2) 21, and P‐selectin glycoprotein ligand‐1 (PSGL‐1) 22. SCARB2 is expressed ubiquitously on the membrane of various cells and tissues 21. As a receptor for all strains of EV71, SCARB2 is capable of mediating viral binding, internalization, and uncoating, and is shown to play a crucial role in the early steps of EV71 infection 23, 24. However, PSGL‐1 expressed on leukocytes has an important role in the tethering and rolling of leukocytes during recruitment of cells from blood vessels to the sites of acute inflammation upon stimulation by infection, but its exact role in nonleukocytes remains unclear 25, 26, 27. A correlation between viral infection and SCARB2 expression appears to be more significant than for PSGL‐1 expression 28. Reports have shown that miR‐127‐5p targets SCARB2 3′ UTR and leads to a significant reduction in both mRNA and protein levels of SCARB2 29. Studies on other viruses have shown that miRNAs can prevent the virus entry by targeting the 3′ untranslated region (UTR) of receptor mRNA to inhibit viral infection. For example, recent studies by Mekky *et al*. 30 revealed that miR‐194 hinders HCV entry through targeting CD81 receptor. miR‐320a and miR‐140 inhibit mink enteritis virus infection by repressing its receptor, feline transferrin receptor 31. miRNAs mediate gene knockdown of cell surface receptors to significantly reduce FMDV infection in cell culture and transgenic suckling mice 32. miR‐23b blocks infections of RV1B through downregulation of its receptor, VLDLR 33. However, although SCARB2 siRNA can significantly reduce EV71 replication 34, whether and how SCARB2 is regulated by cellular miRNAs during EV71 infection are unknown. In the current study, we examined the role of cellular miR‐127‐5p on EV71 infection and found that miR‐127‐5p negatively regulates EV71 replication by suppressing the expression of the EV71 cellular receptor SCARB2. Our results will provide new insights into the inhibitory roles of cellular miR‐127‐5p in EV71 infection and useful information for the identification of novel intervention targets to reduce EV71 infection and pathogenesis.

## Materials and methods

### Cell culture

Vero (derived from African green monkey kidney cells), HeLa (a human epithelial carcinoma cell line), HepG2 (a human hepatoma cell line), 293T (a transformed embryonic kidney cell line expressing SV40 large T antigen), and L929 (mouse fibroblast cells) were grown in Dulbecco's modified Eagle's medium (DMEM; HyClone, Logan, UT, USA) supplemented with 10% or 2% FBS (Gibco, Carlsbad, CA, USA) at 37 °C in a humidified 5% CO_2_ incubator.

### Synthetic oligonucleotides and transfection

Negative control mimics, miR‐127‐5p mimics, negative control inhibitor, and miR‐127‐5p inhibitor were purchased from RiboBio (Guangzhou, China). For transfection, the cells were plated in six‐well plates, grown to 30% confluence, and were transfected with synthetic oligonucleotides using Lipofectamine 3000 (Life Technologies, Carlsbad, CA, USA), according to the manufacturer's instruction.

### RNA isolation and quantitative real‐time PCR

According to the manufacturer's protocol, total RNA was prepared using Trizol reagent (Life Technologies). Reverse‐transcribed cDNA was synthesized using the PrimeScript RT Reagent Kit (TaKaRa, Dalian, China). GAPDH mRNA was used as an endogenous control for the expression of SCARB2 and EV71 VP1 genomic RNA. The primers used were:

SCARB2‐F: 5′‐CCATAGAAGGCATGCACCCA‐3′, SCARB2‐R: 5′‐AGCGCCATGATGATGTAGGG‐3′, EV71VP1‐F: 5′‐GCTCTATAGGAGATAGTGTGAGTAGGG‐3′, EV71 VP1‐R: 5′‐ATGACTGCTCACCTGCGTGTT‐3′, GAPDH‐F: 5′‐TGCACCACCAACTGCTTAGC‐3′, GAPDH‐R: 5′‐GGC ATGGACTGTGGTCATGAG‐3′.

To determine the expression of miRNA in cells, U6 snRNA served as an internal control, and miR‐127‐5p quantitative real‐time PCR (qRT‐PCR) primers were purchased from RiboBio. Real‐time PCR was performed using a standard protocol on an Applied Biosystems 7300 system using SYBR green PCR master mix (Life Technologies) according to the manufacturer's instruction. All reactions were performed in triplicate and relative expression was analyzed using the ΔΔCt method.

### Western blot and antibody reagent

Cells were collected and lysed using RIPA lysis buffer (Santa Cruz, CA, USA) on ice for 20 min and then centrifuged at 12 000 ***g*** for 10 min at 4 °C. Total protein concentrations were determined using the Bicinchoninic Acid (BCA) Protein Assay Kit (Pierce, Rockford, IL, USA). Proteins were analyzed by SDS/PAGE and transferred to polyvinylidene difluoride (PVDF) membranes (Millipore, Billerica, MA, USA). Nonspecific antibody binding was blocked by Odyssey Blocking buffer (LI‐COR Biosciences, Lincoln, NE, USA), and the membranes were incubated with primary antibodies specific for SCARB2 (Santa Cruz Biotechnology, Santa Cruz, CA, USA), EV71 VP1 (Abcam, Cambridge, UK), or GAPDH (Santa Cruz Biotechnology) for 4 h at room temperature (RT). After five washes with PBS‐0.1% Tween‐20 (PBS‐T buffer), the membranes were incubated with IRDye 800 goat anti‐mouse IgG or IRDye 680 donkey anti‐rabbit IgG (LI‐COR Biosciences) at 1 : 10 000 dilution for 1 h and visualized using Li‐COR Odyssey Infrared Imager (LI‐COR Biosciences). Bands were quantified by densitometric analysis using odyssey software.

### Virus titer

Enterovirus 71 Fuyang0805 strain was propagated on Vero cells. Vero cells were grown in 96‐well plates, 24 h before virus infection. EV71 was serially diluted with DMEM supplemented with 2% FBS from 10^3^‐ to 10^10^‐fold, and a dilution of EV71 was added to triplicate wells. The plates were then incubated at 37 °C with 5% CO_2_. The cytopathic effect (CPE) was recorded under the microscope after 3 days. Virus titers were calculated as the 50% tissue culture infectious dose (TCID_50_) using the Reed–Münch method.

### Plasmid constructs

The wild‐type pmirGLO‐SCARB2 3′ UTR reporter plasmid was constructed by inserting the 3′ UTR (nucleotides 270–1240) of SCARB2 into the pmirGLO luciferase vector between the *Xho*I and *Xba*I sites. The SCARB2 3′ UTR was amplified from cDNA using forward primer F (5′ ‐CCGCTCGAGTTGTTGGGTGCTGGTAGCTC‐3′) and the reverse primer R (5′‐GCTCTAGAGGTGTCTCTGCTTCTGGTCC‐3′). The mutant pmirGLO‐SCARB2 3′ UTR construct was generated by inducing a point mutation using the overlap extension PCR method.

### Luciferase assay

For the luciferase assay, 293T cells were plated at a density of 1 × 10^5^ cells per well in 24‐well plates and luciferase reporter plasmids and pLR‐Tk (Promega, Madison, WI, USA) were cotransfected into 293T cells using Lipofectamine 3000 (Life Technologies). After 48 h of incubation, the cells were harvested, washed once with PBS, and lysed with Passive lysis buffer (Promega). Supernatants were collected after 15 min, and centrifuged at 12 000 ***g*** for 30 s. The luciferase activities were measured using a dual‐luciferase reporter system (Promega).

### Immunofluorescence assay

Cells were seeded on glass coverslips, and were infected with EV71 at the indicated time, followed by fixation with 4% paraformaldehyde for 15 min at room temperature. After being washed twice with PBS, the cells were treated with specific primary antibodies against the proteins. The respective secondary antibody was Alexa Fluor 594‐labeled donkey anti‐mouse IgG (Molecular Probes, Life technologies) diluted at 1 : 1000. Images were acquired with an Olympus Fluoview FV10i laser scanning confocal microscope (Tokyo, Japan).

### Flow cytometry

The cells were dispersed with PBS containing 0.02% EDTA, harvested, and fixed in 4% paraformaldehyde. After three washes with PBS, cells were incubated with the SCARB2‐specific antibody (diluted 1 : 100, Santa Cruz, CA, USA) in PBS containing 1% BSA at room temperature for 1 h, followed by incubation with the Alexa Fluor 488‐labeled goat anti‐mouse IgG (Molecular Probes, Life technologies) at 1 : 1000 for 30 min. SCARB2 protein on the cell surface was analyzed by flow cytometry (FACSCalibur, BD Biosciences, San Jose, CA, USA) using a flowjo software (TreeStar Software, San Carlos, CA, USA).

### Statistical analysis

The statistical analyses were performed using prism 5.0 software (GraphPad Software, La Jolla, CA, USA). Differences between experimental groups were statistically evaluated using Student's *t*‐test. *P*‐value of <0.05 was considered statistically significant (*P < 0.05, **P < 0.01, and ***P < 0.001). All the results are presented as means ± SD of at least three independent experiments.

## Results

### miR‐127‐5p inhibits SCARB2 expression by targeting its 3′ UTR

**Figure 1:**
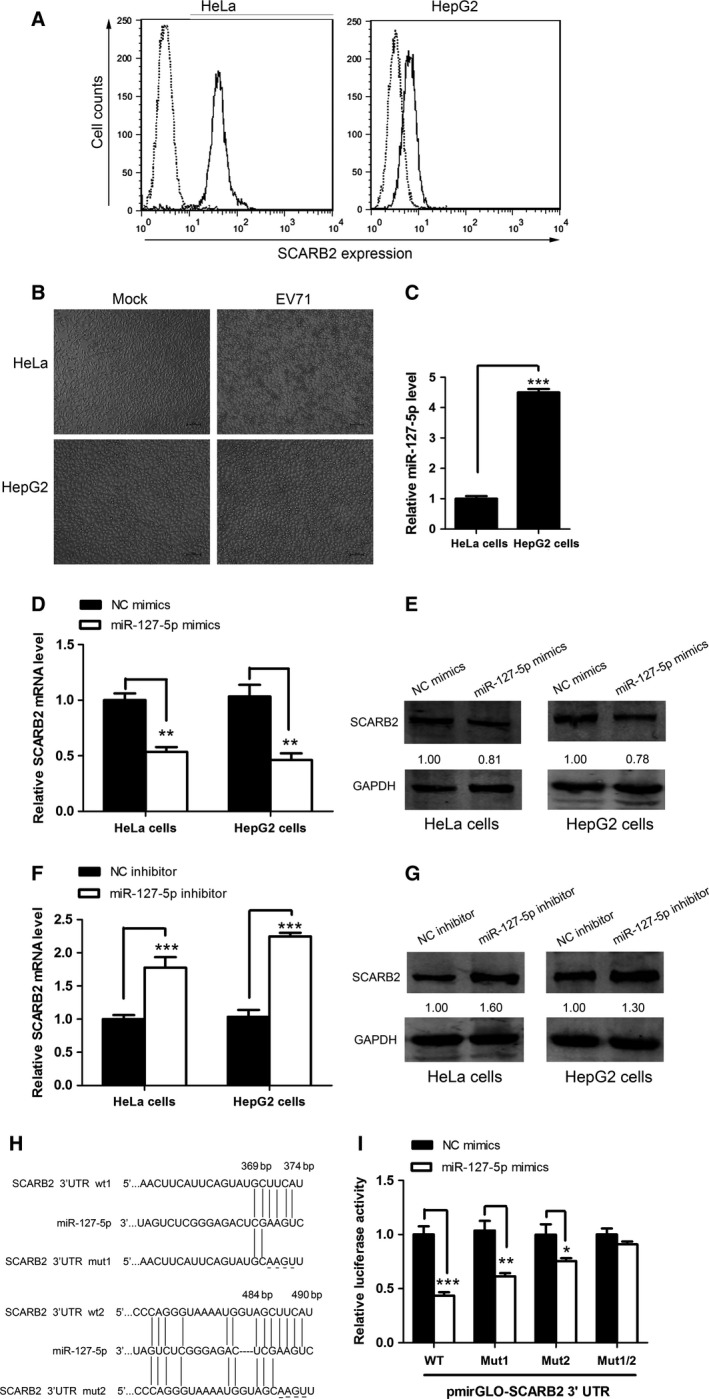
miR‐127‐5p inhibits SCARB2 expression by targeting its 3′ UTR. (A) HeLa and HepG2 cells were stained with the anti‐SCARB2 antibody (solid lines) or normal mouse IgG (dotted lines), and then the surface SCARB2 expression was analyzed by flow cytometry. (B) Susceptibility of HeLa and HepG2 cells to EV71. CPE of HeLa and HepG2 cells infected with EV71. HeLa and HepG2 cells were infected with EV71 at an MOI of 0.1. At 48 h postinfection, these cells were imaged via light microscopy. Scale bar, 100 μm. (C) The cells were lysed and total RNA was extracted. Level of miR‐127‐5p was determined by quantitative real‐time PCR analysis. (D and E) HeLa and HepG2 cells were transfected with miR‐127‐5p mimics or NC mimics (final concentration, 50 nm) for 48 h. (D) The cells were lysed and total RNA was extracted. SCARB2 mRNA level was determined by quantitative real‐time PCR analysis. (E) SCARB2 protein expression in HeLa and HepG2 cells was detected by western blot analysis. The numbers represent the relative density of the band in comparison to the corresponding control normalized to GAPDH. Value of NC mimics treatment is set at 1.00 (100%). (F and G) HeLa and HepG2 cells were transfected with miR‐127‐5p inhibitor or NC inhibitor (final concentration, 100 nm) for 48 h. (F) SCARB2 mRNA level was determined by quantitative real‐time PCR analysis. (G) SCARB2 protein level in HeLa and HepG2 cells was determined by western blot analysis. The numbers denote the relative density of the bands normalized to the control. (H) The illustration of SCARB2 wild‐type or mutant 3′ UTRs containing the putative miR‐127‐5p‐binding sites. (I) Luciferase reporters with SCARB2 3′ UTR wt, SCARB2 3′ UTR mut1, SCARB2 3′ UTR mut2, or SCARB2 3′ UTR mut1/2 were transfected into 293T cells together with miR‐127‐5p mimics or NC mimics. Forty‐eight hours after transfection, the luciferase activity assays were carried out. Data are representative of at least three independent experiments, with each measurement performed in triplicate (mean ± SD of fold‐change). *P < 0.05, **P < 0.01, ***P < 0.001.

Recently, Siebert, *et al*. 29 reported that miR‐127‐5p targeted SCARB2 3′ UTR and reduced the protein expression of SCARB2. As a cellular receptor, SCARB2 can facilitate efficient EV71 infection 35. Firstly, we observed the differential surface expression of SCARB2 on HeLa and HepG2 cells (Fig. 1A), and showed that this differential expression of SCARB2 appears to impact on the susceptibility of HeLa and HepG2 cells to EV71 infection (Fig. 1B). We found that the progression of cytopathic effects in HepG2 cells was slower than that in HeLa cells, suggesting that the efficiency of EV71 infection was correlated with the level of cell surface SCARB2 expression (Fig. 1A,B). Additionally, we also found that HeLa cells expressed a relative lower level of miR‐127‐5p (Fig. 1C). We then investigated if miR‐127‐5p directly suppresses SCARB2 expression by transfecting miR‐127‐5p mimics into both HeLa and HepG2 cells, and then measuring the expression of both SCARB2 mRNA and the protein. We found that miR‐127‐5p mimics significantly reduced both the mRNA level (Fig. 1D) and protein expression of SCARB2 (Fig. 1E), consistent with the previous observations obtained from different cell lines 29. Consistent with above observation, a miR‐127‐5p inhibitor significantly increased mRNA level (Fig. 1F) and protein expression of SCARB2 (Fig. 1G), as compared to the negative control. To further verify that the downregulation of SCARB2 expression is due to targeting by miR‐127‐5p, we performed a bioinformatic search using microRNA (http://www.microrna.org/ to identify putative binding site and identified two seed regions at the 3′ UTR of SCARB2 (nucleotides 369–374 and 484–490) that may bind miR‐127‐5p. In order to demonstrate that the binding sites are specific, we constructed expression plasmids of SCARB2 wild‐type/mutant 3′ UTR containing the putative miR‐127‐5p‐binding sites by cloning into the pmirGLO reporter vector. Mutant reporter constructs were generated with underlined nucleotides indicating the mutated sequences as shown in Fig. 1H. We cotransfected pmirGLO plasmid into 293T cells, along with miR‐127‐5p mimics or negative control mimics. The luciferase activity of SCARB2 3′ UTR wt was significantly reduced in 293T cells treated with miR‐127‐5p mimics, whereas the SCARB2 3′ UTR mut1 or SCARB2 3′ UTR mut2 partly reversed the inhibition of luciferase activity by miR‐127‐5p mimics. Additionally, the luciferase activity in the cells transfected with the SCARB2 3′ UTR mut1/2 vector was unaffected by the miR‐127‐5p mimics (Fig. 1I). This result was in agreement with a previous study showing that miR‐127‐5p had effect on the luciferase activity of pMir‐SCARB2 3′ UTR plasmid 29. In summary, these data suggested that SCARB2 is a direct target of miR‐127‐5p and that its expression is modulated by miR‐127‐5p.

### EV71 infection upregulates miR‐127‐5p expression

**Figure 2:**
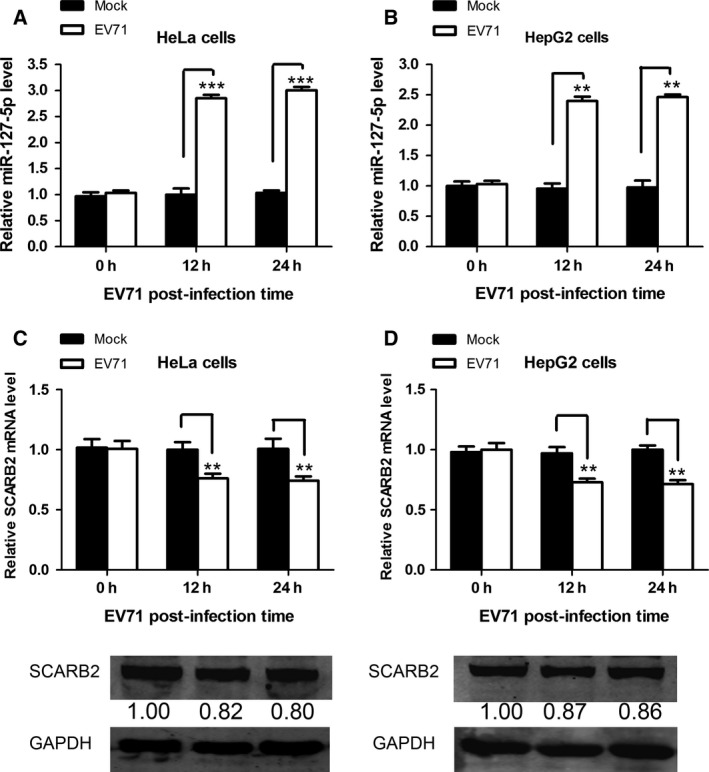
EV71 infection upregulates miR‐127‐5p expression. (A) HeLa and (B) HepG2 cells were infected with EV71 at an MOI of 10. The cells were harvested at 0 h, 12 h, and 24 h postinfection. Total cellular RNA was extracted and the level of miR‐127‐5p was determined by quantitative real‐time PCR. (C) HeLa and (D) HepG2 cells were infected with EV71 at an MOI of 10. The cells were lysed, and mRNA level and protein expression of SCARB2 were determined at the indicated times. The numbers represent the relative density of the bands normalized to the control. Data are representative of at least three independent experiments, with each measurement performed in triplicate (mean ± SD of fold‐change). *P < 0.05, **P < 0.01, ***P < 0.001.

We investigated the expression of miR‐127‐5p in EV71‐infected HeLa cells and found that cellular miR‐127‐5p expression at 12 h and 24 h postinfection was 2.8‐ and 3‐fold higher, respectively, than that of the mock‐infected HeLa cells (Fig. 2A). In EV71‐infected HepG2 cells, the miR‐127‐5p expression at 12 h and 24 h was 2.4‐ and 2.5‐fold higher than those of the mock‐infected cells, respectively (Fig. 2B). Thus, these results demonstrated that EV71 infection upregulated miR‐127‐5p expression. To investigate whether upregulation of the miR‐127‐5p following EV71 infection could affect SCARB2 expression, quantitative real‐time PCR and western blot were performed simultaneously to measure the SCARB2 expression in HeLa (Fig. 2C) and HepG2 cells (Fig. 2D) and, as expected, SCARB2 was slightly, but significantly, downregulated after EV71 infection.

### EV71 gene expression and replication are attenuated by miR‐127‐5p overexpression

**Figure 3:**
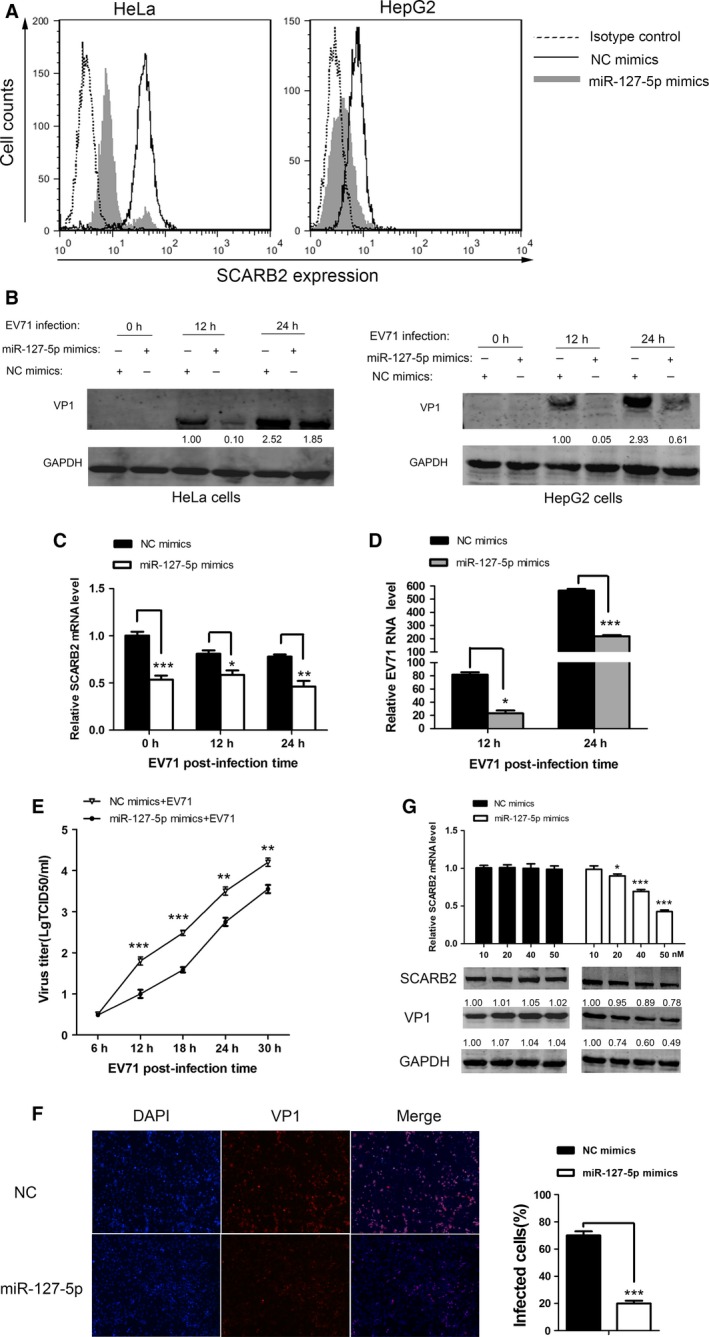
EV71 gene expression and replication are attenuated by miR‐127‐5p overexpression. (A) Flow cytometric analysis was performed to assess the surface SCARB2 level of HeLa and HepG2 cells after 48 h transfection. The cells transfected with NC mimics (final concentration, 50 nm) were stained with the anti‐SCARB2 antibody (solid lines), and transfected with miR‐127‐5p mimics (final concentration, 50 nm) were stained with anti‐SCARB2 antibody (gray region), or the cells were stained with normal mouse IgG (dotted lines, the same result for the cells treating with NC mimics or miR‐127‐5p mimics). (B) HeLa and HepG2 cells were transfected with miR‐127‐5p mimics or NC mimics (final concentration, 50 nm) for 48 h and then infected with EV71 at an MOI of 2.0. The cells were harvested at 12 h and 24 h postinfection, and western blot analysis of the expression levels of EV71 VP1 was performed. The numbers below each band denote the relative density of the bands normalized to the control. (C and D) HeLa cells were transfected with miR‐127‐5p mimics or NC mimics (final concentration, 50 nm) for 48 h and then infected with EV71 (MOI = 2) at the indicated times. (C) SCARB2 mRNA and (D) EV71 VP1 mRNA levels were determined by quantitative real‐time PCR analysis. (E) HeLa cells were transfected with miR‐127‐5p mimics or NC mimics (final concentration, 50 nm) for 48 h and then infected with EV71 (MOI = 0.5) at the indicated times. Culture supernatants were collected at the indicated times and virus was titrated. Virus titers were expressed as the log TCID_50_/mL. (F) HeLa cells were transfected with miR‐127‐5p mimics or NC mimics (final concentration, 50 nm) for 48 h and then infected with EV71 (MOI = 2) at the indicated times. The expression level of VP1 in miR‐127‐5p‐transfected cells infected with EV71. Infected cells were identified by staining with VP1 in red and cell nucleus were counterstained with DAPI (1 mg/mL) in blue. Six sections of the samples were examined at a magnification of 200× using a fluorescence microscope. The quantification represents the average from six different fields. (G) HeLa cells were transfected with different concentrations of miR‐127‐5p mimics or NC mimics for 48 h and infected with EV71 at an MOI of 2.0. At 24 h postinfection, the cells were collected and SCARB2 mRNA level was determined by quantitative real‐time PCR. SCARB2 and VP1 protein levels were determined by western blot and normalized to GAPDH. The numbers denote the relative density of the bands normalized to the control. Data are representative of at least three independent experiments, with each measurement performed in triplicate (mean ± SD of fold‐change). *P < 0.05, **P < 0.01, ***P < 0.001.

Earlier studies showed that SCARB2 siRNA treatment inhibited EV71 infection 34, 36. Similarly, our results have confirmed that miR‐127‐5p could suppress the SCARB2 expression (Fig. 1A,B). To further substantiate whether modulation of miR‐127‐5p expression would affect the surface expression of SCARB2, we transfected miR‐127‐5p mimics into both HeLa and HepG2 cells, and then analyzed by flow cytometry. The result suggested that upregulation of the miR‐127‐5p level led to a decrease in the surface expression of SCARB2 (Fig. 3A). Therefore, it is of interest to evaluate the effect of miR‐127‐5p on EV71 replication. For this purpose, miR‐127‐5p was overexpressed by miRNA mimics transfection and its effect on EV71 replication was assessed by measuring EV71 VP1 expression, the viral RNA level, viral titer, and percentage of infected cells. EV71 VP1 protein decreased in both HeLa and HepG2 cells overexpressing miR‐127‐5p, as compared to the negative control cells (Fig. 3B). The levels of SCARB2 mRNA (Fig. 3C) and EV71 RNA (Fig. 3D) in cells transfected with miR‐127‐5p mimics were significantly reduced. The EV71 titers at various times postinfection were also significantly suppressed by miR‐127‐5p overexpression, as compared to the control (Fig. 3E). Immunofluorescence staining of viral VP1 showed that the percentage of infected cells in cells overexpressing miR‐127‐5p was reduced by nearly 50%, as compared to the control, at 24 h postinfection (Fig. 3F). When HeLa cells were transfected with increasing concentrations of miR‐127‐5p mimics (10, 20, 40, and 50 nm), followed by EV71 infection, both SCARB2 mRNA and protein levels, as well as EV71 VP1 protein, were inhibited as a function of the dose of miR‐127‐5p mimics (Fig. 3G). These results showed that miR‐127‐5p overexpression downregulated SCARB2 expression and thus significantly reduced EV71 infection.

### Suppression of endogenous miR‐127‐5p enhances EV71 replication

**Figure 4:**
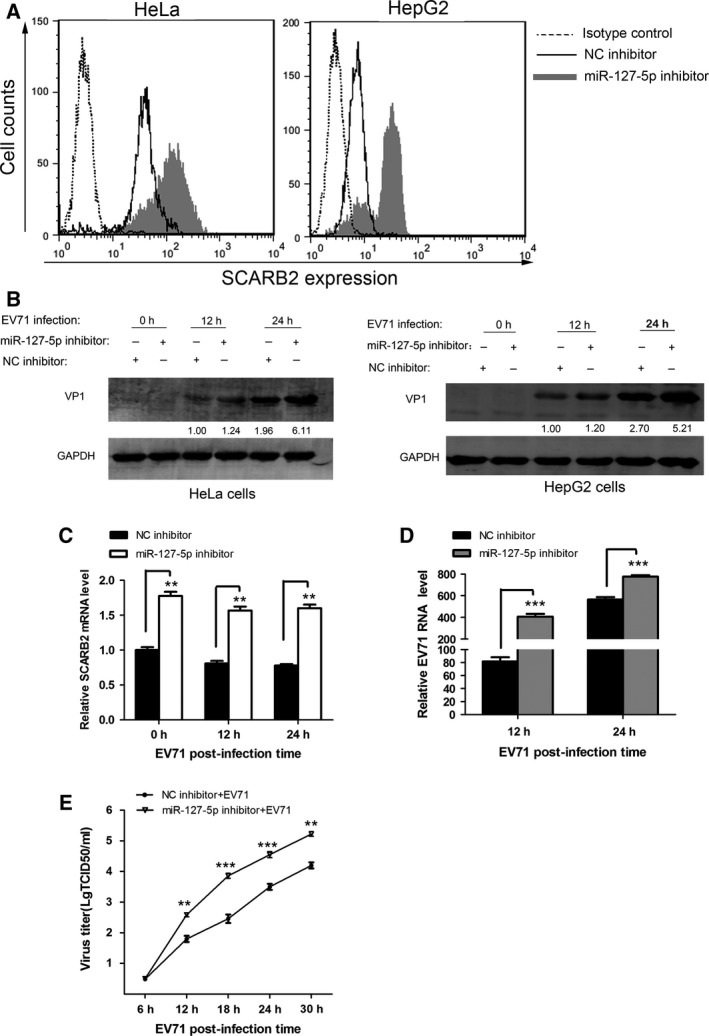
Suppression of endogenous miR‐127‐5p enhances EV71 replication. (A) Flow cytometric analysis was used to assess the surface SCARB2 level of HeLa and HepG2 cells after 48 h transfection. The cells transfected with NC inhibitor (final concentration, 100 nm) were stained with the anti‐SCARB2 antibody (solid lines), and transfected with miR‐127‐5p inhibitor (final concentration, 100 nm) were stained with anti‐SCARB2 antibody (gray region), or the cells were stained with normal mouse IgG (dotted lines, the same result for the cells treating with NC inhibitor or miR‐127‐5p inhibitor). (B) HeLa and HepG2 cells were transfected with miR‐127‐5p inhibitor or NC inhibitor (final concentration, 100 nm) for 48 h and then infected with EV71 at an MOI of 2.0. The cells were harvested at 12 h and 24 h postinfection, and western blot analysis of the expression levels of EV71 VP1 was performed. The numbers below each band denote the relative density of the bands normalized to the control. (C–E) HeLa cells were transfected with miR‐127‐5p inhibitor or NC inhibitor for 48 h and then infected with EV71 (MOI = 2.0) at the indicated times. (C) SCARB2 mRNA and (D) EV71 VP1 mRNA levels were determined by quantitative real‐time PCR analysis. (E) HeLa cells were transfected with miR‐127‐5p inhibitor or NC inhibitor for 48 h and then infected with EV71 (MOI = 0.5) at the indicated times. Culture supernatants were collected at the indicated times and virus was titrated. Virus titers were expressed as the log TCID_50_/mL. Data are representative of at least three independent experiments, with each measurement performed in triplicate (mean ± SD of fold‐change). *P < 0.05, **P < 0.01, ***P < 0.001.

Next, we performed experiments to determine whether inhibition of endogenous miR‐127‐5p expression affects EV71 replication. A chemically synthesized oligonucleotide with sequence complementary to endogenous miR‐127‐5p was used to inhibit endogenous miR‐127‐5p expression. Such inhibitor oligonucleotides have been shown to sequester intracellular miRNAs and to inhibit their activity in the RNA interference pathway 37, 38. Interestingly, transfection of miR‐127‐5p inhibitor increased the surface SCARB2 expression (Fig. 4A). Moreover, HeLa and HepG2 cells transfected with the miR‐127‐5p inhibitor or NC inhibitor were subsequently infected with EV71, and EV71 VP1 expression, SCARB2 mRNA level, the viral RNA level, and viral titers were determined, respectively. The expression of EV71 VP1 protein (Fig. 4B) in HeLa and HepG2 cells was upregulated by the inhibition of endogenous miR‐127‐5p, as shown by western blot analysis. Consistently, the levels of SCARB2 mRNA (Fig. 4C) and EV71 RNA (Fig. 4D) were also higher in cells transfected with miR‐127‐5p inhibitor than in the negative control cells. We also found that the EV71 titer was significantly higher in cells treated with miR‐127‐5p inhibitor than that in untreated cells (Fig. 4E). In conclusion, these results demonstrated that inhibition of endogenous miR‐127‐5p led to enhancement of EV71 replication.

### miR‐127‐5p does not influence intracellular replication of EV71

**Figure 5:**
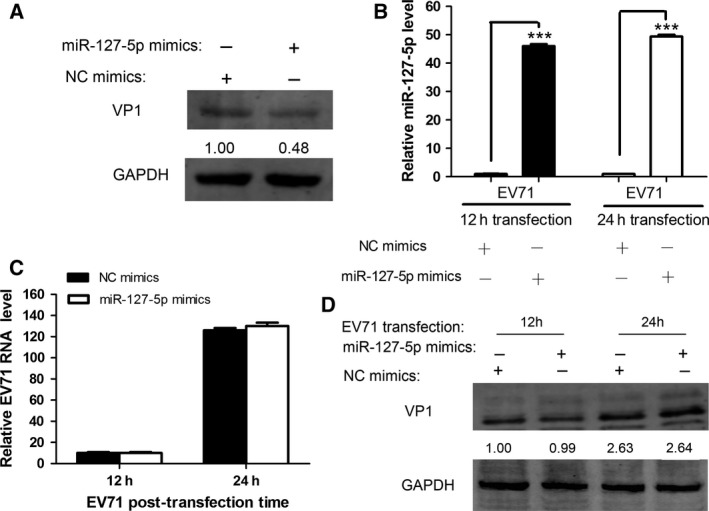
miR‐127‐5p does not influence intracellular replication of EV71. (A) HeLa cells were transfected with miR‐127‐5p mimics or NC mimics (final concentration, 50 nm) for 48 h and the cells were infected by EV71 at an MOI of 10 in PBS containing 0.05% EDTA for 1 h at 4 °C. The cells were washed, lysed and EV71 VP1 protein level was determined by western blot and normalized to GAPDH. The numbers denote the relative density of the bands normalized to the control. (B–D) L929 cells were cotransfected with EV71 genomic RNA and miR‐127‐5p or NC mimics for 12 h and 24 h. Levels of miR‐127‐5p (B) and EV71 RNA (C) were determined by quantitative real‐time PCR analysis. (D) EV71 VP1 level was determined by western blot and normalized to GAPDH. The numbers denote the relative density of the bands normalized to the control. Data are representative of at least three independent experiments, with each measurement performed in triplicate (mean ± SD of fold‐change). ***P < 0.001.

We postulate that mechanistically miR‐127‐5p downregulates cellular receptor SCARB2 and thus prevents the EV71 attachment to and entry into the cells. To further investigate the mechanism of miR‐127‐5p inhibition, we conducted a virus attachment analysis to determine if viral attachment is affected by the miR‐127‐5p‐mediated down‐modulation of SCARB2. The result showed that miR‐127‐5p significantly reduced the amount of VP1, as compared to the control cells (Fig. 5A). The above results suggested that miR‐127‐5p blocked entry of EV71 by inhibiting expression of the viral receptor SCARB2, resulting in the reduction of viral attachment.

Previous studies reported that viral genomic RNA is directly targeted by host miRNAs 39, 40. In order to rule out the possibility that EV71 genomic RNA is targeted by miR‐127‐5p in our research, studies were carried out to determine if EV71 genomic RNA contains potential target sequences for miR‐127‐5p. It had been demonstrated that hSCARB2, but not mScarb2, bound efficiently to EV71 41. Mouse L929 cells are not susceptible to EV71 infection and allow only inefficient EV71 infection, because they lack the cellular receptor 21, 24, 35. In this study, we used L929 cells to investigate whether EV71 replication initiated by transfection of infectious viral genomic RNA is affected by miR‐127‐5p. High level of miR‐127‐5p was detected in miR‐127‐5p‐transfected L929 cells, suggesting that the miR‐127‐5p was efficiently incorporated intracellularly (Fig. 5B). In the L929 cells transfected with EV71 genomic RNA, although increasing amounts of viral RNA were detected, no inhibitory effect of miR‐127‐5p was observed (Fig. 5C). In addition, EV71 VP1 expression showed no difference between the cells overexpressing miR‐127‐5p and the control cells (Fig. 5D). These results demonstrated that miR‐127‐5p suppressed infection of EV71 by downregulating the expression of its major cellular receptor, SCARB2, rather than impairing the intracellular viral replication.

## Discussion

In recent years, morbidity and mortality due to EV71 infection have increased, whereas the underlying pathogenic mechanisms remain elusive, and intervention and therapeutic approaches are still limited. MicroRNAs serve as multifunctional regulators, and the significance of miRNAs in virus–host interactions is becoming evident. A growing number of studies show that host miRNAs can regulate viral replication by altering the expression of host genes required for viral response 30, 42, 43 or by directly targeting viral genomic RNAs 39, 40, 44. To date, few studies have already explored the effects of miRNAs in EV71 infection; for example, miR‐296‐5p targets the EV71 genome to suppress viral replication 39; miR‐23b inhibits EV71 replication through downregulation of VP1 protein 45; and our study showed that miR‐30a inhibited EV71 replication by modulating EV71‐induced autophagy 46. In this report, we provided evidence on the effect of cellular miR‐127‐5p on SCARB2 expression and its inhibitory activity on EV71 infection. SCARB2 is considered to be a crucial EV71 receptor 23.

SCARB2 plays critical roles in viral attachment, entry, and uncoating, and it can promote EV71 infection 24. Recently, a study demonstrated that human miR‐127‐5p significantly downregulated SCARB2 protein level by directly targeting SCARB2 mRNA 29. As an important receptor for EV71, reduction in SCARB2 may prevent viral entry during primary infection. Thus, it is plausible to assume that miR‐127‐5p may impact EV71 infection by regulating SCARB2 expression. In the current study, we first analyzed the influence of miR‐127‐5p mimics (Fig. 1E) or inhibitor (Fig. 1F) on the total cellular SCARB2 level by western blotting. Because SCARB2 is a lysosomal protein, and the majority of the protein is located in the lysosome and endosomes, these results will be difficult to accurately reflect the change of surface SCARB2. Therefore, we performed the flow cytometric analysis of the cell surface SCARB2 expression, and the data indicated that overexpression of miR‐127‐5p significantly inhibited the surface SCARB2 expression (Fig. 3A), while inhibition of endogenous miR‐127‐5p significantly elevated the surface SCARB2 (Fig. 4A). Consistently, overexpression of miR‐127‐5p or inhibition of endogenous miRNA‐127‐5p resulted in repressed (Fig. 3B) or enhanced (Fig. 4B) production of EV71, respectively. Results from dual‐luciferase assay validated that miR‐127‐5p targeted SCARB2 and thus inhibited protein translation of SCARB2. Studies with SCARB2 mutants and the inability of miR‐127‐5p in inhibition of EV71 replication in L929 cells (Fig. 5B–D) suggested that this miR‐127‐5p‐mediated downregulation of SCARB2 was specific and miR‐127‐5p did not target at intracellular or viral sequences.

As miR‐127‐5p‐mediated downregulation of SCARB2 likely impact only on the uninfected cells for their susceptibility to EV71 infection, it is likely that reduced infection is mainly resulted from decreased viral attachment to the cells with miR‐127‐5p‐mediated SCARB2 downregulation. Our evidence that VP1 was reduced by more than 50% in cells overexpressing miR‐127‐5p and infected with EV71 for 1 h at 4 °C illustrated that the downregulated SCARB2 led to the reduced viral attachment. Downregulation of SCARB2 by miR‐127‐5p may be of significance for host defense to the EV71 as the binding of virus to the host cell is the initial step for viral entry; therefore, transient downregulation of cell surface molecules may be an effective strategy to block and reduce viral transmission. As several reports have shown 47, 48, 49, siRNAs can inhibit viral infection through various different mechanisms and can be used to control virus diseases *in vivo*.

Overall, we identified miR‐127‐5p as a novel EV71 entry inhibitor through targeting SCARB2 expression. To our knowledge this is the first study describing the role of microRNAs in regulating SCARB2 expression during EV71 infection. Endogenous miRNAs may possess advantages over siRNAs on safety and fewer side effects. The current study provides a new perspective for miRNA‐mediated antiviral effects and contributes to the better understanding of host–virus interaction mechanisms.

## Author contributions

ZW conceived and supervised the study. CF designed and performed experiments. YF and DC provided new reagents and analyzed data. HW, AS, and NZ interpreted the data. LZ and LC provided new reagents. CF wrote the manuscript. ZW made manuscript revisions.
